# Interface-Driven Assembly of Pentacene/MoS_2_ Lateral Heterostructures

**DOI:** 10.1021/acs.jpcc.1c06661

**Published:** 2022-01-10

**Authors:** Francesco Tumino, Andi Rabia, Andrea Li Bassi, Sergio Tosoni, Carlo S. Casari

**Affiliations:** † Dipartimento di Energia, Politecnico di Milano, via G. Ponzio 34/3, Milano I-20133, Italy; ‡ Dipartimento di Scienza dei Materiali, Università di Milano-Bicocca, via Roberto Cozzi 55, 20125 Milano, Italy

## Abstract

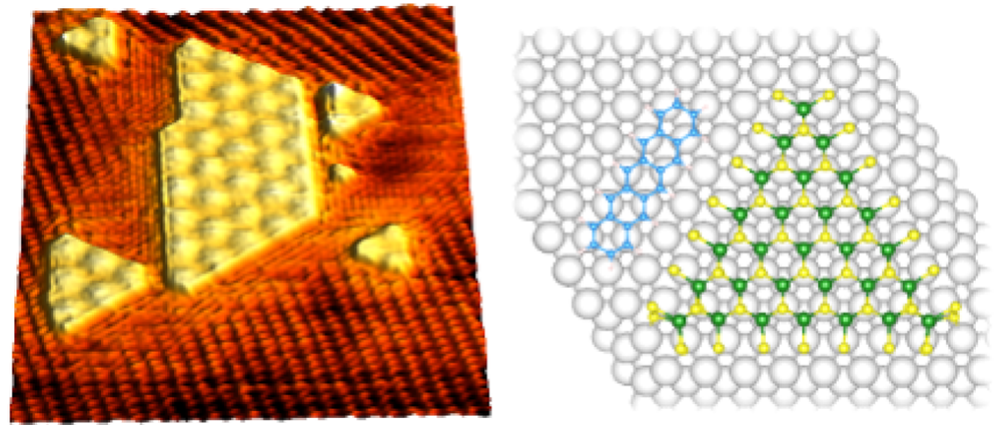

Mixed-dimensional van der Waals heterostructures formed by molecular assemblies and 2D materials provide a novel platform for fundamental nanoscience and future nanoelectronics applications. Here we investigate a prototypical hybrid heterostructure between pentacene molecules and 2D MoS_2_ nanocrystals, deposited on Au(111) by combining pulsed laser deposition and organic molecular beam epitaxy. The obtained structures were investigated in situ by scanning tunneling microscopy and spectroscopy and analyzed theoretically by density functional theory calculations. Our results show the formation of atomically thin pentacene/MoS_2_ lateral heterostructures on the Au substrate. The most stable pentacene adsorption site corresponds to MoS_2_ terminations, where the molecules self-assemble parallel to the direction of MoS_2_ edges. The density of states changes sharply across the pentacene/MoS_2_ interface, indicating a weak interfacial coupling, which leaves the electronic signature of MoS_2_ edge states unaltered. This work unveils the self-organization of abrupt mixed-dimensional lateral heterostructures, opening to hybrid devices based on organic/inorganic one-dimensional junctions.

## Introduction

van der Waals heterostructures (vdWHs) have emerged as novel low-dimensional systems of great potential for the development of ultrathin devices with tailored properties. In the most common approach, distinct 2D dangling-bond-free layers are used as building blocks to form vertically stacked vdWHs. The structural and electronic variety resulting from the combination of different 2D crystals can be remarkably enriched by adding dimensionality as a further parameter in the choice of single components, thus extending the vdWH concept beyond the framework of *all-2D* heterostructures. Combining materials with different dimensionalities in the so-called *mixed-dimensional* vdWH^1^ has recently begun to attract interest for the possibility to significantly broaden the range of properties, functionalities, and potential applications of vdWHs. In particular, 0D–2D vdWHs formed by small organic molecules and 2D inorganic semiconductors provide a novel platform to design and study innovative proof-of-concept optoelectronic devices.^1−6^ A prominent example is the vertical stacking of pentacene and MoS_2_ in a mixed-dimensional organic/inorganic vdWH which forms a gate-tunable photovoltaic junction with antiambipolar characteristics and long-lived charge-separated states.^3,5,6^

In parallel to the development of vertical mixed-dimensional vdWHs, the possibility to fabricate lateral (i.e., in-plane) hybrid heterostructures has been recently demonstrated.^7−10^ Lateral vdWHs can form one-dimensional heterojunctions, enabling the development of atomically thin circuitry. However, the nanoscale assembly of low-dimensional materials into a functional lateral heterostructure poses important challenges, such as (i) the development of bottom-up synthesis methods providing molecular-scale control of the assembly structure and (ii) the detailed investigation of the lateral heterointerface at the molecular level. Addressing these issues requires the controlled synthesis of model heterostructures under ideal conditions, e.g., using ultrahigh vacuum (UHV) growth approaches, and their in situ nanoscale characterization by high-resolution techniques, such as scanning tunneling microscopy (STM) and spectroscopy (STS). This experimental approach has recently led to the study of lateral heterostructures between molecules and 2D materials, such as graphene,^7^ MoS_2_,^8^ and borophene.^9^

In this work, we focus on the combination of MoS_2_ and pentacene—which is presently the most performing molecule in organic electronics—in a mixed-dimensional lateral heterostructure. We used the Au(111) surface as a template for growing single-layer (SL) MoS_2_ crystals and pentacene assemblies by pulsed laser deposition (PLD) and organic molecular beam epitaxy (OMBE), respectively, under UHV conditions. In situ STM–STS, corroborated by dispersion-corrected density functional theory (DFT+D) calculations, shows the formation of in-plane pentacene–MoS_2_ heterostructures on the Au(111) surface. The molecular arrangement at the interface with MoS_2_ is driven by the interaction between pentacene and MoS_2_ edges. However, such an interaction does not affect the MoS_2_ electronic edge states, which reveal their metallic character in spatial resolved STS. Our findings provide the first molecular-scale experimental observation of pentacene/MoS_2_ lateral heterostructures, whose structural and electronic abruptness opens to atomically thin vdWH devices with one-dimensional heterojunctions.

## Methods

### Sample Preparation

All experiments were conducted in an UHV system composed of three interconnected chambers for PLD, OMBE, and STM/STS characterization. Au(111)/mica substrates (Mateck) were cleaned by cycles of Ar^+^ sputtering (1 keV) and annealing at 700 K. MoS_2_ was deposited by PLD on freshly prepared Au(111) at room temperature (RT) and subsequently annealed at 730 K for 30 min. The PLD process was optimized to obtain a submonolayer coverage of well-ordered single-layer MoS_2_ nanocrystals. Briefly, a rotating MoS_2_ target (Testbourne) was ablated by KrF laser pulses (248 nm wavelength, 10 ns pulse duration) at a repetition rate of 1 pulse per second and a laser fluence of 2 J/cm^2^. The desired MoS_2_ coverage on the substrate—placed at 3 cm from the target—was achieved with six laser pulses. Pentacene (Sigma Aldrich, 98% purity) was deposited on MoS_2_/Au(111) samples at RT from an effusion cell (Dr. Eberl–MBE Komponenten) heated at 428 K. A mild annealing at 350 K was performed after deposition.

### Scanning Tunneling Microscopy/Spectroscopy

STM/STS measurements were acquired at RT with an Omicron microscope, using homemade electrochemically etched W tips. Typical measurement parameters were in the range 0.5–2 V for bias voltage and 0.1–0.3 nA for set-point current (the specific values for the reported STM images are stated in the captions). The differential conductivity (*dI*/*dV*) was measured using a lock-in amplifier applying a modulation voltage of 30 mV_rms_ at 6 kHz. *I*(*V*) and *dI*/*dV*(*V*) curves have been acquired simultaneously in open-feedback-loop conditions, using 1.5 V and 0.2 nA as set-point parameters. Line-mode STS was performed to investigate the lateral interface between different materials. Since data were acquired at RT, we had to compensate for the thermal drift to minimize the uncertainty in the tip position along the line.

### Computational Methods

DFT calculations are carried out with the code VASP.^11,12^ Core electrons are described with the projector-augmented wave scheme,^13,14^ while H(1s), C(2s,2p), Mo(4d,4p,5s), S(3s,3p), and Au(5d,6s) are treated explicitly. We adopt the Perdew, Burke, and Ernzerhof (PBE)^15^ functional. The long-range dispersion is accounted for, recurring to the D3 semiempirical scheme and the Becke–Johnson damping.^16,17^ The plane-wave basis set is expanded within a kinetic energy cutoff of 400 eV. Convergence thresholds of 10^–5^ eV (electronic loop) and 10^–2^ eV/Å (ionic loop) are adopted. The sampling of the reciprocal space is reduced to the Γ point only. Dipole and quadrupole corrections to the total energy are applied along the nonperiodic direction. In order to avoid spurious interactions between replicas of the slab models, a vacuum region of at least 15 Å is included in the supercells. The monolayer (ML) MoS_2_/Au(111) interface is described by superposing a 10 × 10 MoS_2_ on a 11 × 11 Au(111) supercell as in our previous work.^18^ The same 11 × 11 Au(111) supercell is also adopted for the simulation of MoS_2_ islands as well as for pentacene adsorption.

## Results and Discussion

We prepared pentacene/MoS_2_ heterostructures following a two-step procedure: first, we synthesized single-layer (SL) MoS_2_ islands on Au(111) by pulsed laser deposition (PLD); second, after having observed the MoS_2_ growth by STM, we deposited pentacene by organic molecular beam epitaxy (OMBE).

**Figure 1:**
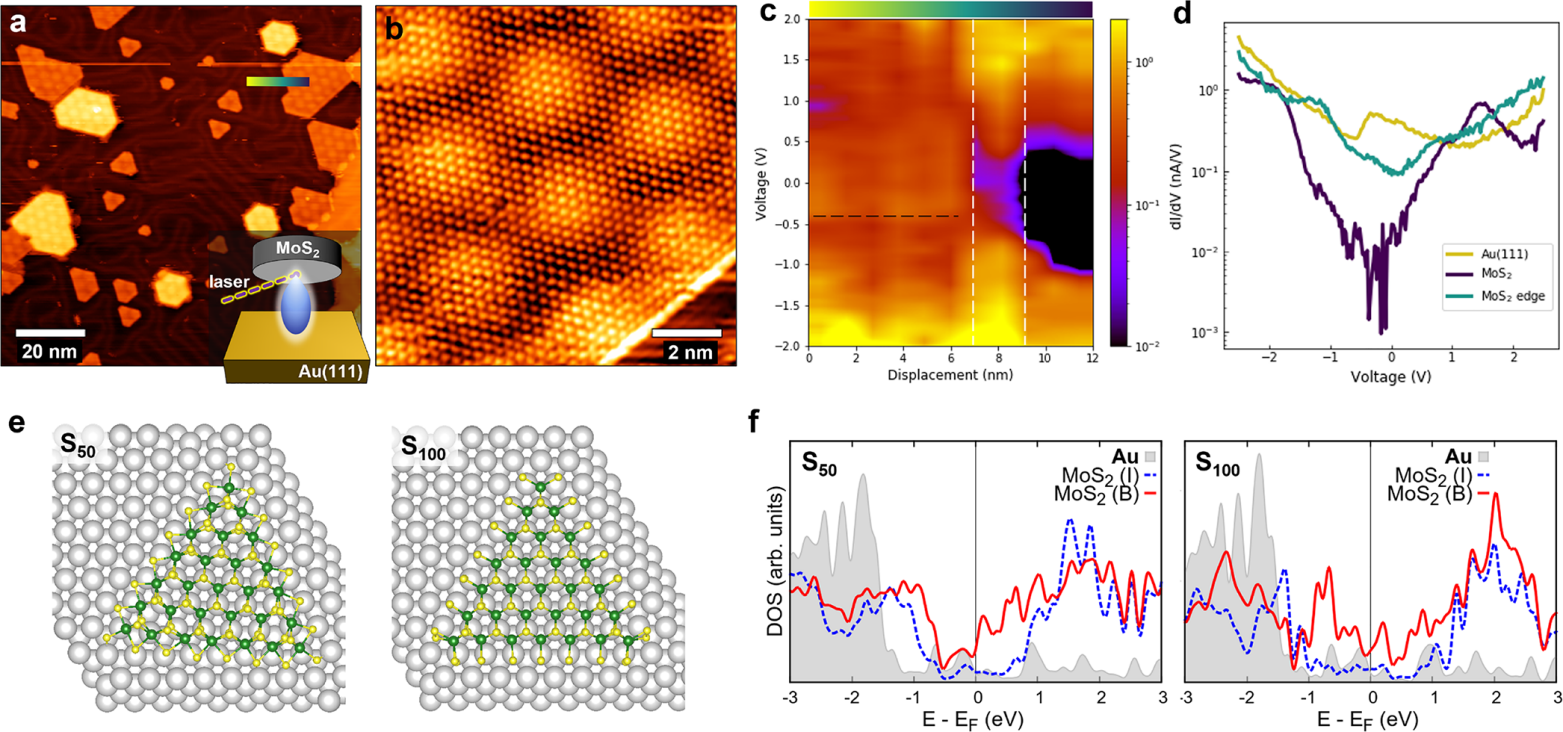
STM/STS data of MoS_2_/Au(111) before pentacene deposition. (a) Large-scale STM image of MoS_2_ islands on Au(111) (1.2 V, 0.3 nA). Inset: schematic of the PLD process, in which intense laser pulses hit the MoS_2_ target, producing a plasma plume of ablated species which condenses on the surface. (b) Atomic resolution STM image showing the MoS_2_ lattice and the Moiré pattern (0.5 V, 0.3 nA). (c) Color map of STS data obtained along a line crossing the Au–MoS_2_ interface (reported in (a)). The color gradient represents *dI*/*dV* (nA/V) in log scale. The black horizontal line indicates the onset of the Au(111) surface state. The white vertical lines indicatively mark the interface region. (d) Point spectroscopy data acquired on the Au(111) (gold line), edge (green), and center (purple) of MoS_2_ islands. (e) Structural models of S_50_ (left) and S_100_ (right) islands. (f) Calculated PDOS of S_50_ (left) and S_100_ (right) islands. The gray shaded area is Au PDOS, and the red solid line and the blue dashed ones are MoS_2_ PDOS at the border and at the island center, respectively.

We will start by discussing the main features of MoS_2_ on Au(111), observed before pentacene deposition. Following our previous work,^19^ we developed a PLD procedure to grow SL MoS_2_ islands on Au(111): briefly, we deposit MoS_2_ precursors by ablating a stoichiometric target with a few laser pulses (inset of Figure 1a) and then anneal the sample at 730 K to favor the crystallization of MoS_2_ structures. The large-scale STM image in Figure 1a shows SL MoS_2_ islands on Au(111) obtained with six laser pulses. The growth of MoS_2_ islands lifts the surface reconstruction, causing the herringbone ridges to follow a more distorted pattern compared to the regular zigzag pattern of clean Au(111). The apparent height of MoS_2_ islands is ∼2 Å, relative to the gold terrace. The presence of higher islands, having a brighter color in Figure 1a, has been previously reported^19,20^ and associated with SL MoS_2_ growing on top of monatomic Au islands, which emerge from Au terraces as a possible consequence of stress release mechanisms induced by MoS_2_ growth (see SI, Figure S1). Atomic resolution images (Figure 1b) show the surface S atoms of the MoS_2_ lattice (∼3.16 Å periodicity) and the hexagonal Moiré pattern (∼32 Å periodicity) generated by the mismatch with Au(111). The Moiré superlattice can be interpreted as a 10/11 MoS_2_/Au coincidence with no rotational mismatch, as reported in previous works.^18^

We investigated the local electronic properties by means of STS measurements acquired on different surface regions. The color map in Figure 1c shows the differential conductivity (*dI*/*dV*) acquired along a line from Au to the center of a MoS_2_ island. The bare Au region is characterized by the onset of the Au(111) surface state at 0.4 eV below the Fermi level (horizontal black line). At the opposite end of the line, an ∼1.5 eV band gap characterizes the MoS_2_ region, in agreement with previous STS measurements of MoS_2_/Au(111).^21−23^ The border of MoS_2_ islands (between the two vertical white lines) shows a finite density of states (DOS) in the gap region, suggesting the influence of edge electronic states. Figure 1d shows the *dI*/*dV* curves of the three regions, obtained by averaging several point spectra acquired on the Au(111) (gold line), edge (green), and center (purple) of MoS_2_ islands. The presence of intragap states localized at the MoS_2_ edges is evidenced by the metallic character of the corresponding local DOS (green line), in contrast to the semiconducting behavior of the inner part of MoS_2_ islands. This observation agrees with previous reports of metallic edge states in MoS_2_ nanocrystals on Au(111).^24−26^

To analyze in more detail the observed electronic properties, we conducted DFT calculations on MoS_2_ islands supported on a four-layer Au(111) slab. The islands have triangular shapes, and their borders are cut along the (10 1̅0) crystallographic direction, which was proven to be thermodynamically favorable by means of DFT calculations.^27,28^ Each side is composed of seven molybdenum atoms, which is a size range where the most important chemical properties are reasonably converged.^29^ However, one should be aware that the islands considered in the simulations are remarkably smaller than the ones grown experimentally, and all considerations on the stability and preferential shape of these objects may depend on their size, even though this should not influence the interaction with pentacene. We considered two types of terminations, S_50_ (Figure 1e, left) and S_100_ (Figure 1e, right), differing in the loading of S atoms at the border, a parameter that is sensitive to the chemical environment of the deposition. The S_50_ model (Mo_45_S_99_) displays single S atoms bridging two peripheral Mo atoms and pointing downward to the Au substrate, saturating 50% of the dangling bonds at the border Mo atoms. The S_100_ model (Mo_45_S_126_) displays ending S_2_ dimers in the MoS_2_ lattice positions, saturating 100% of the Mo dangling bonds. During the relaxation, S_50_ assumes a rotated position relative to the metal substrate, while S_100_ remains almost aligned. In the inner region of the islands, the MoS_2_/Au interfacial distance resembles the one observed in the case of an extended MoS_2_ monolayer (ML) (2.6 Å),^18^ while in corner regions there are closer S–Au contacts (2.4 Å). Both models display an interesting electronic feature, revealed in the projected DOS (PDOS) plots reported in Figure 1f: at variance from ML MoS_2_/Au(111) (see SI, figure S2), where the PDOS projected on the supported film orbitals displays a gap comparable to the free-standing case, here the region around the Fermi level is populated by several states located at the island borders. This supports the assignment of the local DOS observed by STS at the MoS_2_/Au interface as edge states. As noted before, S_100_ islands are aligned with the Au substrate, as the islands observed experimentally. Therefore, in our growth conditions the formation of islands terminated like the S_100_ is favored against the S_50_. This suggests that the growth occurs in a nondeficient S environment, in agreement with the fact that the PLD process is approximately stoichiometric.

**Figure 2:**
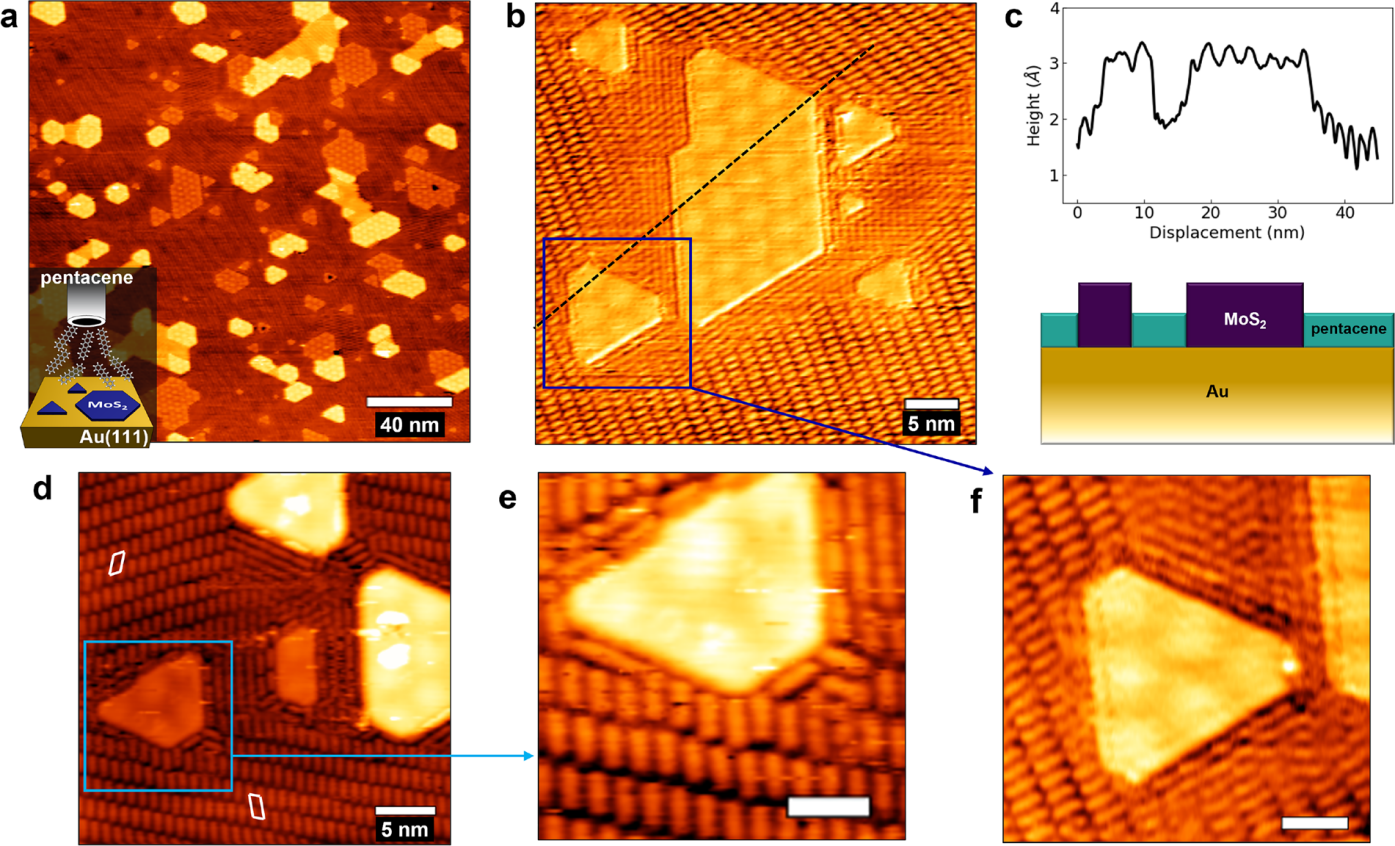
STM images of pentacene/MoS_2_ on Au(111). (a) Large-scale STM image showing SL MoS_2_ islands surrounded by pentacene molecules (1.2 V, 0.17 nA). Inset: schematic of pentacene deposition by evaporation onto previously prepared MoS_2_/Au(111). (b) STM image showing the self-assembly of pentacene molecules around the border of MoS_2_ islands (0.8 V, 0.15 nA). (c) Top: Line profile along the dashed line in (b). Bottom: schematic of the sample topography. (d) STM image showing the perturbation of pentacene self-assembly due to the presence of MoS_2_ islands (2.2 V, 0.3 nA). The white parallelograms label the unit cells of twin molecular lattices far from MoS_2_ edges. (e),(f) STM images showing at higher resolution the MoS_2_ islands framed in (d) and (b), respectively. Scale bars: 3 nm. (e) 2.2 V, 0.3 nA. (f) 0.8 V, 0.15 nA.

After having studied the MoS_2_/Au(111) system, we deposited pentacene by molecular evaporation onto the sample at RT. The deposition was followed by a mild annealing at 350 K, to favor desorption of possible impurities resulting from the evaporation process. Figure 2a shows a large-scale STM image taken after deposition: the Au surface not occupied by MoS_2_ is now entirely covered by a monolayer self-assembly of pentacene molecules which overlays the herringbone reconstruction without lifting it. Higher-resolution images (Figure 2b) show that the Moiré pattern on MoS_2_ islands is not affected by the deposition, a sign that pentacene molecules do not lie on top of MoS_2_ nor intercalate between MoS_2_ and Au. Far from MoS_2_ islands, the molecular arrangement follows the same patterns which can be observed on clean Au(111), i.e., without MoS_2_. Indeed, from preliminary investigations, we observed that pentacene can form various ordered monolayer domains, coexisting on the Au(111) surface, where molecules are arranged in geometrically distinct molecular lattices. Some representative STM images of pentacene/Au(111) are reported in the SI (Figure S3) and are in good agreement with previous literature.^30,31^ The preference for pentacene to grow only on the bare Au surface rather than on top of MoS_2_ leads to the formation of lateral (i.e., in-plane) interfaces between pentacene and SL MoS_2_ islands. The situation is depicted in Figure 2c, where the line profile (top) along the dashed line in Figure 2b is accompanied by a schematic of the sample topography (bottom). Interestingly, Figure 2b also shows that the presence of MoS_2_ islands perturbs the ordered arrangement of pentacene molecules, which assemble in different configurations close to MoS_2_. Therefore, the packing geometry of the molecular assembly is locally altered, and in some cases, as in the central part of Figure 2b, it looks like the pentacene surface density increases near MoS_2_ borders. This effect combined with increased disorder near the border possibly causes a lower resolution in STM imaging, which prevents us from a more quantitative analysis of the perturbed lattice. Also, we notice that the perturbation induced by MoS_2_ borders affects in some cases a relatively large area of the molecular assembly (as in the central part of Figure 2b), whereas somewhere else (as in the upper border of the large MoS_2_ islands in Figure 2b) it may affect only the molecules directly facing MoS_2_ termination. We can attribute this difference to the presence of MoS_2_ borders of different islands relatively close to each other in the central part of Figure 2b, which contribute to a more extended alteration of the ordered molecular arrangement. In Figure 2d, where two twin molecular lattices can be identified (unit cells labeled in white), we observe that the molecules change their orientation in proximity to MoS_2_ borders, thus losing registry with the ordered domains. A closer look at the MoS_2_ border regions reveals a tendency for pentacene molecules to align parallel to MoS_2_ edges. A distinction has to be noted with reference to Figure 2d: the two MoS_2_ nanocrystals with darker contrast grow directly on the Au terrace, whereas the other two brighter MoS_2_ nanocrystals grow on top of Au islands, as described previously (see also Figure S1). Close to the brighter islands, pentacene is in lateral contact with the step edge of the Au island supporting MoS_2_. In this case, the molecular alignment is governed by the interaction with the Au step edge, rather than MoS_2_ terminations. In the following, we will not consider such a situation and focus only on the lateral interface between pentacene and MoS_2_, e.g., formed around the darker islands in Figure 2d. Such an interface can be observed at higher resolution in the STM images of Figure 2e,f, which show that most molecules align parallel to the MoS_2_ border.

**Figure 3:**
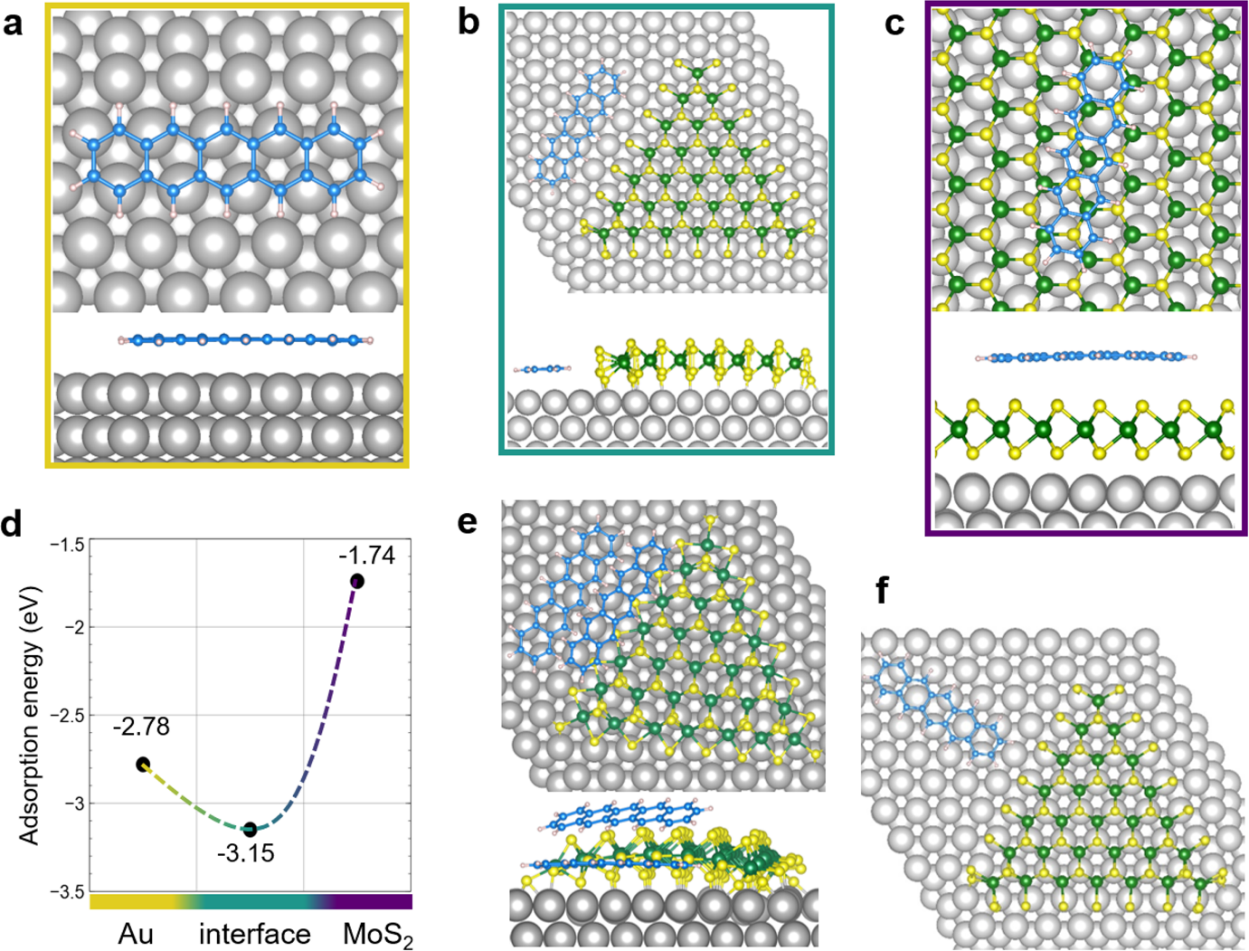
Top (above) and side (below) views of the DFT structures for pentacene adsorbed (a) on Au(111), (b) at the MoS_2_–S_100_/Au border (parallel configuration), and (c) on the monolayer MoS_2_/Au. (d) Pentacene adsorption energy for (a), (b), and (c) cases. (e) Adsorption of two pentacene molecules at the MoS_2_–S_50_/Au interface. (f) Pentacene adsorbed at the MoS_2_–S_100_/Au border (orthogonal configuration).

In order to shed light on the nature and relative strength of the interactions established by pentacene with the gold substrate and the MoS_2_ borders, we performed DFT calculations on the three models reported in Figure 3a–c. In Figure 3a, pentacene is adsorbed on the Au(111) surface in a hollow configuration, previously reported as the global minimum in a computational study,^32^ yielding an adsorption energy of −2.78 eV relative to the clean substrate and the molecule in the gas phase. This value is very close to previous dispersion-corrected DFT calculations.^33^ It can be observed that the molecule lies almost perfectly flat on the metal surface. In Figure 3b, the pentacene molecule is adsorbed at the border of the MoS_2_–S_100_ island. The molecule is aligned parallel to the island border and is slightly tilted relative to the Au substrate. This site is remarkably more favorable for pentacene adsorption compared to Au(111), and the adsorption energy is now −3.15 eV, indicating that both the gold surface and island border atoms cooperate in binding the molecule. A very similar adsorption energy, −3.11 eV, is yielded for an analogous configuration at the MoS_2_–S_50_ island border (Figure S4a), indicating that the chemical composition of the island termination has little effect on the stabilization of pentacene, which can be justified by the dispersive and chemically nonspecific nature of the interaction. In Figure 3c, the pentacene adsorbed on the MoS_2_ single layer supported on Au is shown. Here the adsorption energy (−1.74 eV) is smaller compared to that reported for the Au surface or the MoS_2_ interface. It is worth noting that, if pentacene is adsorbed on free-standing MoS_2_ islands (Figure S5), either on top of the flat terraces or at the borders (with little changes related to the adsorption configuration and the chemical nature of the termination), adsorption energies similar to the monolayer (in the range between −1.3 eV and −1.7 eV) are obtained. This proves that the molecule stabilization at the MoS_2_/Au border, as highlighted in Figure 3d, is an interface effect. However, even though the arrangement of Figure 3c is less favorable compared to the interface region (Figure 3b) and the clean Au(111) surface (Figure 3a), the calculations indicate that pentacene can be adsorbed also on the inner region of the MoS_2_ islands, a fact which, at first glance, is in contrast with the STM images (Figure 2). In fact, this result is not surprising, if one considers the universal and size-dependent nature of van der Waals forces, implying that pentacene should bind to the surface of MoS_2_ islands as well. However, one should also consider that molecules deposited at RT, like in the present work, will have enough thermal energy to evolve toward the thermodynamically favored sites of adsorption. The trend in adsorption strength emerging from DFT calculations (MoS_2_/Au interface > Au > MoS_2_) is also supported by recent temperature-programmed desorption measurements available for pentacene on Au(111) and basal MoS_2_(001), also showing a larger zero-coverage extrapolated desorption energy on Au (2.2 eV)^34^ compared to MoS_2_ (1.2 eV).^35^ In general, the presently adopted DFT-D3 approach is thus robust in depicting stability trends in weakly bound interfaces. However, the absolute adsorption energies may be overestimated with respect to accurate experimental calorimetric measurements, as also recently discussed for condensed aromatic molecules on Au(111).^33^ To further investigate this aspect, we started from the structure analogous to the one reported in Figure 3b for the S_50_ termination and put a second pentacene molecule tilted at the island border (Figure 3e). This yielded an adsorption energy of −2.47 eV per pentacene molecule, indicating that the adsorption of further pentacene molecules in the border region is still more favorable than covering the MoS_2_ islands. We conclude that, even if a full pentacene coverage was reached at the MoS_2_/Au interface, the creation of multilayer pentacene adducts in this region would be more favorable compared to covering the inner MoS_2_ region. This fully accounts for the evidence from STM measurements reported in Figure 2. Finally, we checked the stability of a pentacene molecule adsorbed at the MoS_2_/Au interface orthogonally relative to the island border (Figure 3f). In this case, an adsorption energy of −2.89 eV is obtained, confirming thus that the parallel configuration is more favorable (see also SI, Figure S4b).

**Figure 4:**
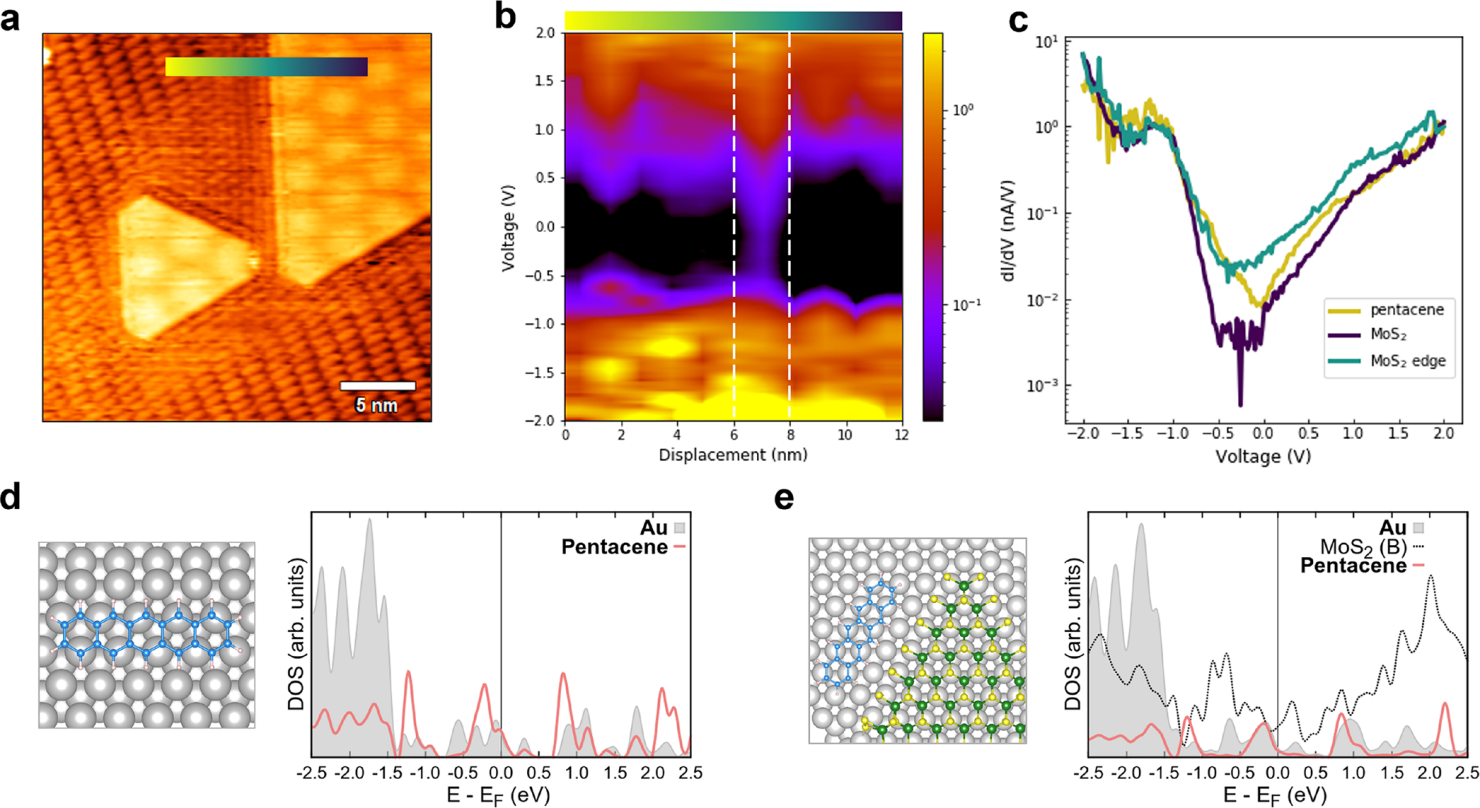
(a) STM image of pentacene/MoS_2_ on Au(111) showing the interfacial regions of the lateral heterostructure (0.8 V, 0.15 nA). (b) Color map of STS data obtained along a line crossing the pentacene/MoS_2_ interface (reported in (a)). The color gradient represents *dI*/*dV* (nA/V) in log scale. The white vertical lines indicatively mark the interface region. (c) Point spectroscopy data acquired on the pentacene layer far from the MoS_2_ borders (gold line), edge (green), and center (purple) of MoS_2_ islands. Calculated PDOS for (d) pentacene/Au and (e) pentacene/MoS_2_–S_100_/Au. Corresponding structural models are reported on the left. The gray shaded area is Au PDOS; red solid lines are pentacene PDOS; and the black dashed line in (e) is MoS_2_ PDOS at the border.

To assess the electronic properties of the pentacene/MoS_2_ system, we performed STS measurements across the heterointerface. *dI*/*dV* curves were acquired as the tip was moved from pentacene to MoS_2_ along a straight line (reported in Figure 4a). The data are shown in the color map of Figure 4a. The DOS in the pentacene region is characterized by a small gap approximately from −0.5 to 0.5 eV, in agreement with reported STS of isolated pentacene molecules on Au.^36^ The interface region is characterized by the electronic features of MoS_2_ edge states, closing the energy gap at around ∼7 nm, as also shown by the corresponding *dI*/*dV* curve in Figure 4c (green line). The band gap is then restored in the MoS_2_ region (∼8–12 nm). The spectral feature at about −1.2 eV is shown by all the spectra in Figure 4c and is not present in the MoS_2_ spectrum of Figure 1d: it could be due to the influence of tip states during the acquisition of STS measurements. The observation of MoS_2_ edge states suggests that pentacene is not strongly bound to MoS_2_ terminations, which then retains its original electronic structure.

The calculated PDOS, reported in Figures 4d and 4e, provide some useful information. First of all, one can notice that, for pentacene on Au(111), the peak related to the molecule HOMO lies at −0.33 eV relative to the Au Fermi energy, while the LUMO is at +0.89 eV. This is in reasonable agreement with ultraviolet photoelectron spectroscopy (UPS) measurements on the pentacene/Au interface (−0.5 eV for HOMO and +1.0 eV for LUMO).^37^ The situation at the MoS_2_/Au interface is different: besides the small positive shift of the pentacene HOMO, in the energy range corresponding to the MoS_2_ band gap, there are many interfacial states related to the bonding between the unsaturated atoms at the MoS_2_ borders and the metal substrates,^29^ at variance relative to that observed for an infinite MoS_2_ film supported on Au(111).^18^ Simulations corroborate the experimental observation that the lateral assembly of pentacene molecules does not affect significantly the electronic structure of MoS_2_ borders. This behavior determines the abrupt transition of the local DOS across the lateral interface.

## Conclusions

In conclusion, we reported a molecular-resolution investigation of lateral pentacene/MoS_2_ nanoheterostructures, supported by Au(111). STM/S measurements and DFT calculations showed that interfacing pentacene molecules with single-layer MoS_2_ result in a unique self-assembly where molecules are aligned along MoS_2_ edges. The local electronic DOS undergoes an abrupt transition across the in-plane interface, characterized by the contribution of MoS_2_ metallic edges states. Our findings shed light on the nanoscale physics of the system, highlighting the stabilizing role of MoS_2_ terminations in driving the interface assembly of pentacene molecules. This work shows the potential of molecular-scale investigation of hybrid lateral heterostructures, opening to further investigations and future engineering of atomically thin mixed-dimensional devices.

## Supporting Information Available

Additional STM images of MoS_2_/Au(111) and pentacene/Au(111), computational results on MoS_2_ DOS, and pentacene adsorption (PDF)
