# Supplementary material for: Interface-Driven Assembly of Pentacene/MoS2 Lateral Heterostructures
Source: J Phys Chem C Nanomater Interfaces. 2022 Jan 10;126(2):1132–9. doi: 10.1021/acs.jpcc.1c06661 (PMC8785183; doi:10.1021/acs.jpcc.1c06661)
Supplement: Supplementary file 1 — jp1c06661_si_001.pdf [file jp1c06661_si_001.pdf]

SUPPLEMENTARY INFORMATION

Interface-driven Assembly of Pentacene/MoS_2 Lateral Heterostructures

Francesco Tumino[1,*], Andi Rabia[1], Andrea Li Bassi[1], Sergio Tosoni[2] and Carlo S. Casari[1]

[1]Dipartimento di Energia, Politecnico di Milano, via G. Ponzio 34/3, I-20133 Milano, Italy
[2]Dipartimento di Scienza dei materiali, Università di Milano-Bicocca, via Roberto Cozzi 55, 20125 Milano, Italy

*Corresponding author: francesco.tumino@polimi.it

MoS_2 on Au(111): additional STM data

Figure S1a shows a large-scale STM image of single-layer MoS_2 nanocrystals on Au(111). As better shown in figure S1b, some islands have a brighter contrast in STM images. Such islands are ~2.3 Å higher than the others, independently on the bias voltage. This height difference is consistent with a monoatomic step of Au(111) (figure S1c). As reported in previous works,[1,2] the presence of these islands can be attributed to SLNCs growing on top monoatomic Au islands, which emerge from Au terraces as a possible consequence of stress release mechanisms induced by MoS_2 growth. The topographic representation in the bottom panel of figure S1c schematically represents this structural effect and qualitatively reproduces the line profile in the upper panel.

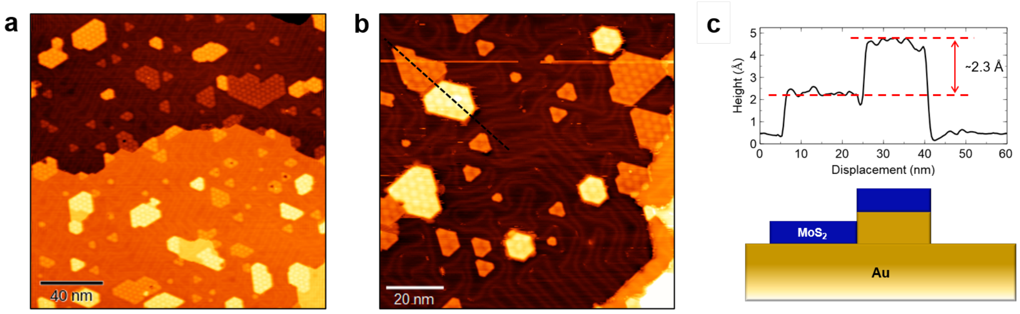

Figure S1: (a)-(b) STM images of MoS_2 islands on Au(111). (c) Top: Line profile taken along the black dashed line in (b). Bottom: Schematic representation of the corresponding topographic profile.

MoS_2 on Au(111): DOS of monolayer MoS_2

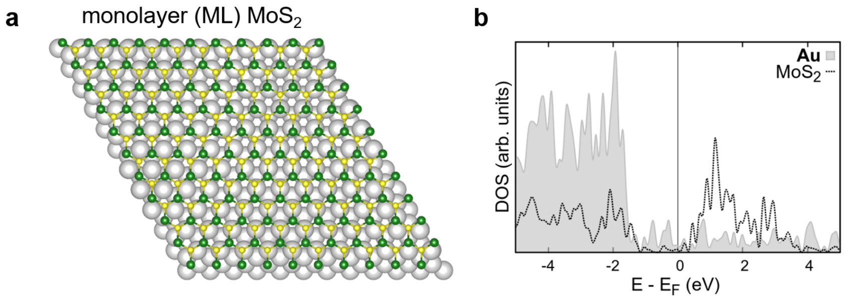

Figure S2: (a) Structural model of a monolayer (ML) MoS_2 film on Au(111). (b) Calculated DOS of ML MoS_2/Au(111).

Pentacene on Au(111)

Previous literature reports[3,4] show that pentacene on Au(111) can form several incommensurate lattices in the submonolayer–monolayer range (at least 12 distinct ordered phases were reported), where the molecules lie down parallel to the surface.

Accordingly, we observed different self-assembly geometries coexisting on the substrate surface, as shown for instance by the large-scale STM image in figure S3, where different pentacene lattices are separated by the dashed line. Figures S3b-d show three of such lattices at high resolution. The typical elongated morphology of single pentacene molecules (whose $C_{22}H_{14}$ structure is depicted in the inset of figure S3a) can be easily distinguished in the three STM images. From the analysis of 2D Fourier transforms, we identified the lattice unit cells, which are schematically reported below the corresponding STM image along with the structural details.

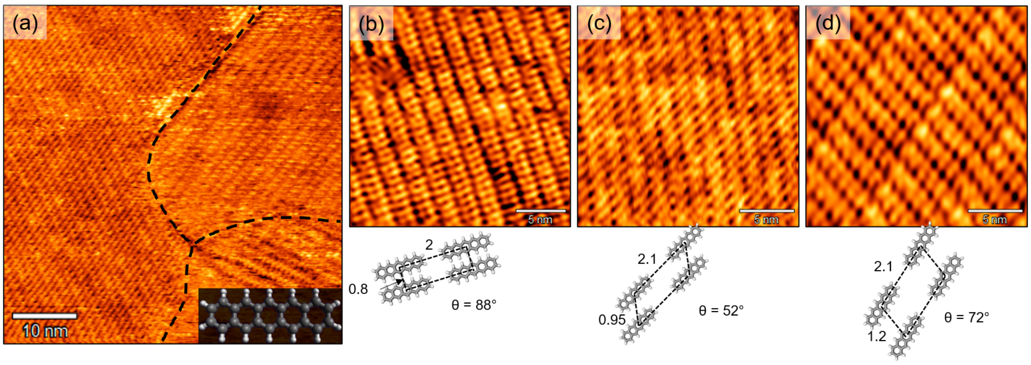

Figure S3: (a) Large-scale STM image of pentacene molecules on Au(111). The dashed line indicates the boundary between different self-assembly domains. Inset: ball-and-stick structure of a pentacene molecule. (b)-(d) STM images of three different molecular lattices. Below each image the corresponding lattice unit cell is reported: the cell sides are expressed in nm, θ is the acute angle between them.

Adsorption of pentacene at the MoS_2–S_{50} edge and on freestanding MoS_2 islands

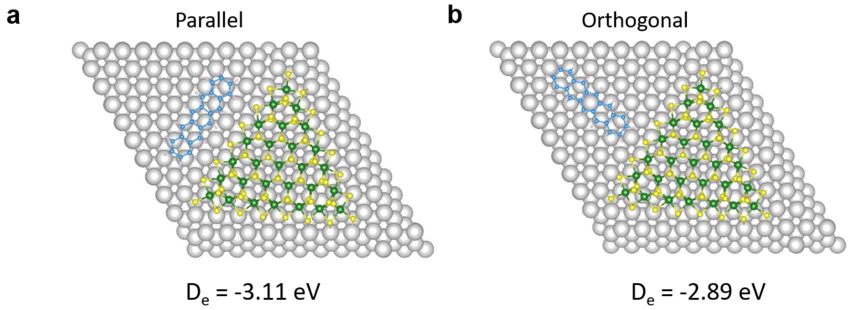

Figure S4: Top views and adsorption energy (D_e, eV) for pentacene adsorbed at the border of S_{50} MoS_2/Au(111) in parallel (a) and orthogonal (b) configuration.

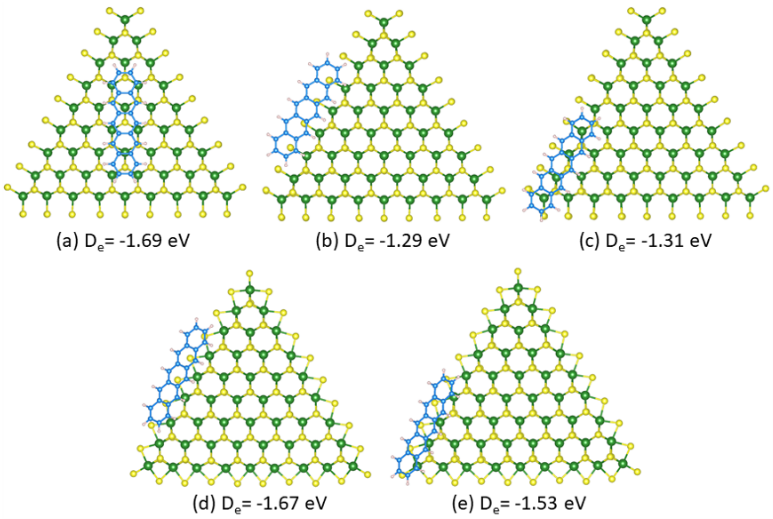

Figure S5: Top views and adsorption energy (D_e, eV) for pentacene adsorbed on freestanding MoS_2 islands. (a) S_{100}, inner region; (b) S_{100}, border; (c) S_{100}, corner; (d) S_{50}, border; (e) S_{50}, corner.

References

[1] Francesco Tumino, Carlo S Casari, Matteo Passoni, Valeria Russo, and Andrea Li Bassi. Pulsed laser deposition of single-layer MoS_2 on Au(111): from nanosized crystals to large-area films. *Nanoscale Adv.*, 1(2):643–655, 2019.

[2] Signe S Grønborg, Søren Ulstrup, Marco Bianchi, Maciej Dendzik, Charlotte E Sanders, Jeppe V Lauritsen, Philip Hofmann, and Jill A Miwa. Synthesis of epitaxial single-layer MoS_2 on Au(111). *Langmuir*, 31(35):9700–9706, 2015.

[3] CB France, PG Schroeder, JC Forsythe, and BA Parkinson. Scanning tunneling microscopy study of the coverage-dependent structures of pentacene on Au(111). *Langmuir*, 19(4):1274–1281, 2003.

[4] JH Kang and X-Y Zhu. Layer-by-layer growth of incommensurate, polycrystalline, lying-down pentacene thin films on Au(111). *Chem. Mater.*, 18(5):1318–1323, 2006.
